# A detailed gene expression study of the *Miscanthus* genus reveals changes in the transcriptome associated with the rejuvenation of spring rhizomes

**DOI:** 10.1186/1471-2164-14-864

**Published:** 2013-12-09

**Authors:** Adam Barling, Kankshita Swaminathan, Therese Mitros, Brandon T James, Juliette Morris, Ornella Ngamboma, Megan C Hall, Jessica Kirkpatrick, Magdy Alabady, Ashley K Spence, Matthew E Hudson, Daniel S Rokhsar, Stephen P Moose

**Affiliations:** Energy Biosciences Institute, Institute for Genomic Biology, University of Illinois Urbana, 1206 West Gregory Drive, Urbana, IL 61801 USA; Crop Sciences, University of Illinois, AW-101 Turner Hall, 1102 S. Goodwin Avenue, Urbana, IL 61801 USA; Energy Biosciences Institute, University of California, 135 Energy Biosciences Building, Berkeley, CA 94720 USA; Department of Molecular and Cell Biology, Life Sciences Annex, University of California, Berkeley, CA 94720 USA; Department of Plant Biology, Edward R. Madigan Laboratory, University of Illinois, 1201 West Gregory Drive, Urbana, IL 61801 USA; DOE Joint Genome Institute, 2800 Mitchell Drive, Walnut Creek, CA 94598 USA; Bio Architecture Lab Inc, 604 Bancroft Way, Berkeley, CA 94710 USA; Department of Plant Biology, University of Georgia, Athens, GA 30602 USA; The Procter & Gamble Company, Mason Business Center, 8700 Mason Montgomery Road Box 513, Mason, OH 45040-9462 USA

**Keywords:** Transcriptome, Miscanthus, Illumina, Short read sequencing, RNA sequencing, Development

## Abstract

**Background:**

The *Miscanthus* genus of perennial C4 grasses contains promising biofuel crops for temperate climates. However, few genomic resources exist for *Miscanthus*, which limits understanding of its interesting biology and future genetic improvement. A comprehensive catalog of expressed sequences were generated from a variety of *Miscanthus* species and tissue types, with an emphasis on characterizing gene expression changes in spring compared to fall rhizomes.

**Results:**

Illumina short read sequencing technology was used to produce transcriptome sequences from different tissues and organs during distinct developmental stages for multiple *Miscanthus* species, including *Miscanthus sinensis*, *Miscanthus sacchariflorus*, and their interspecific hybrid *Miscanthus* × *giganteus*. More than fifty billion base-pairs of *Miscanthus* transcript sequence were produced. Overall, 26,230 *Sorghum* gene models (i.e., ~ 96% of predicted *Sorghum* genes) had at least five *Miscanthus* reads mapped to them, suggesting that a large portion of the *Miscanthus* transcriptome is represented in this dataset. The *Miscanthus* × *giganteus* data was used to identify genes preferentially expressed in a single tissue, such as the spring rhizome, using *Sorghum bicolor* as a reference. Quantitative real-time PCR was used to verify examples of preferential expression predicted via RNA-Seq. Contiguous consensus transcript sequences were assembled for each species and annotated using InterProScan. Sequences from the assembled transcriptome were used to amplify genomic segments from a doubled haploid *Miscanthus sinensis* and from *Miscanthus* × *giganteus* to further disentangle the allelic and paralogous variations in genes.

**Conclusions:**

This large expressed sequence tag collection creates a valuable resource for the study of *Miscanthus* biology by providing detailed gene sequence information and tissue preferred expression patterns. We have successfully generated a database of transcriptome assemblies and demonstrated its use in the study of genes of interest. Analysis of gene expression profiles revealed biological pathways that exhibit altered regulation in spring compared to fall rhizomes, which are consistent with their different physiological functions. The expression profiles of the subterranean rhizome provides a better understanding of the biological activities of the underground stem structures that are essentials for perenniality and the storage or remobilization of carbon and nutrient resources.

**Electronic supplementary material:**

The online version of this article (doi:10.1186/1471-2164-14-864) contains supplementary material, which is available to authorized users.

## Background

*Miscanthus* is a perennial C4 grass that belongs to the *Andropogoneae* tribe within the Poaceae family, which includes important agricultural crops for food and fuel such as sugarcane, sorghum, and maize. Following their introduction into the Western world in the 1930s [1], members of the *Miscanthus* genus are now grown as ornamental crops in many regions of the United States due to their characteristically robust growth and attractive late-season inflorescence.

The *Miscanthus* genus consists of approximately fifteen species, most of which are either diploids or tetraploids [2]. The grass is an obligate outcrosser with a large, highly repetitive 2.5 Gbp (giga base pairs) genome that is distributed across nineteen chromosomes [3, 4]. Natural hybridization events between the two most predominant *Miscanthus* species, *M. sinensis* and *M. sacchariflorus*, have been reported [5, 6]. Ribosomal DNA evidence suggests that the large statured, cold tolerant, sterile triploid hybrid *M. × giganteus* (3*n* = 57) is the result of a natural hybridization event between a diploid *M. sinensis* (2*n* = 38) and a tetraploid *M. sacchariflorus* (4*n* = 76) [2, 4, 7].

Plants of the *Miscanthus* genus, especially *Miscanthus* × *giganteus*, have generated interest as a source of lignocellulosic biomass for the bioenergy industry. Although *Miscanthus* has been of horticultural interest for some time, it essentially remains a genus of wild species. Genetic selections for the genus have largely concentrated on traits desirable to the horticultural and landscaping industry; there have been few focused breeding efforts targeting traits that would enhance the potential of *Miscanthus* as a perennial bioenergy feedstock. The availability of molecular tools for *Miscanthus* will accelerate improvement of biofuel-centric traits in *Miscanthus*. Recent advances in *Miscanthus* genomics have enabled the construction of complete genetic maps for *M. sinensis*[8–10]. These genetic maps revealed a recent allotetraploidization event in Miscanthus in which pairs of homeologous chromosomes show extensive synteny to the *Sorghum bicolor* genome, with a single chromosome fusion accounting for the nineteen linkage groups.

Deep sequencing technologies applied to gene discovery through transcriptome sequencing has efficiently increased genetic information for many non-model plant organisms such as barley, grape, wheat, and lodgepole pine [11–15]. Importantly, the high degree of sequence similarity and genome organization between *Miscanthus* and *Sorghum* make *Sorghum bicolor* a suitable reference genome sequence for the analysis of the *Miscanthus* transcriptome [4, 9, 10]. A preliminary study of dormant *Miscanthus × giganteus* rhizomes was used to assess variation among available *Miscanthus × giganteus* accessions [16], but a comprehensive catalog of expressed sequences in the *Miscanthus* genus is not yet available. We report here high-depth sequencing of expressed mRNAs from a variety of *M. × giganteus* tissues as well as multiple accessions of *M. sinensis* and one accession of *M. sacchariflorus*. The data generated enable a robust assembly of the *Miscanthus* transcriptome with demonstrated utility in the analysis of changes in gene expression and evolution of genic sequences within the genus.

## Results and discussion

### Sequencing the *Miscanthus* transcriptome

**Figure 1:**
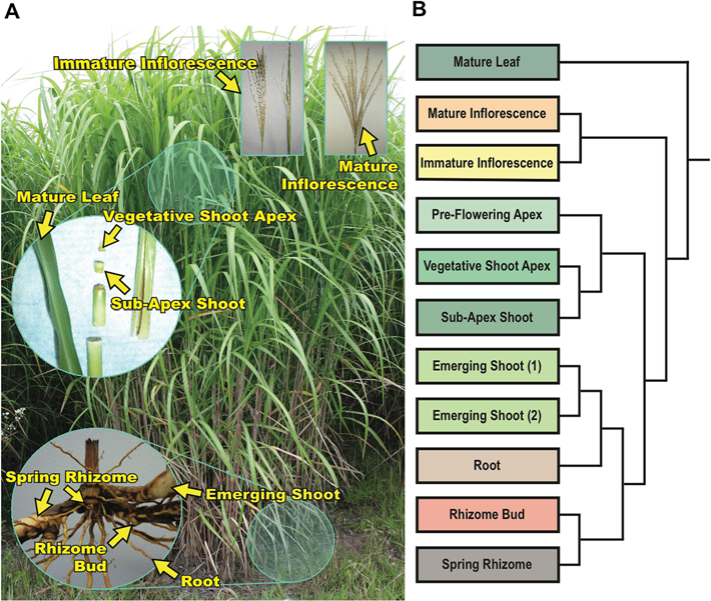
**Sampled** ***Miscanthus × giganteus*** **tissue types and relatedness of EST profiles using** ***Sorghum bicolor*** **gene models as references.** Panel **A** is an image identifying many of the *M. × giganteus* tissues used in this study. Panel **B** displays the relatedness of the sequenced tissue types by hierarchical clustering of the expression profiles using Manhattan distance and complete linkage.

To obtain a global overview of gene expression in *Miscanthus* and maximize transcript representation of the genus, 767 million expressed sequence tags (ESTs) were generated from eight *Miscanthus* accessions using Illumina’s sequencing by synthesis technology (Table 1, Figure 1A). To this end, we sequenced six *M. sinensis* accessions, one *M. sacchariflorus*, and the Illinois clone of *Miscanthus × giganteus*. For *M. × giganteus*, RNA-Seq libraries were constructed from eleven organs at a variety of developmental stages and sequenced separately (Figure 1A). The *M. sacchariflorus* and *M. sinensis* libraries were either generated from a mixture of tissues pooled together or from expanding leaves with both immature and mature tissues (Table 1).

**Table 1:** ***Miscanthus*** **RNA-Seq libraries sequenced for this study**

Miscanthus accessions	Tissue	Total bases
		(Billion base pairs)
*Miscanthus* x *giganteus* ‘Illinois clone’	RO^2^, RZ^1 and 3^, RB^2^, ES^1 and 2^, VA^1^, SA^1^, ST^2^, PA^2^, II^1^, MI^2^, ML^1^, FR^3^	41.50
*Miscanthus sacchariflorus* ‘Golf Course’	Mixed	6.54
*Miscanthus sinensis* ‘White Kaskade’	Mixed	4.32
*Miscanthus sinensis* ‘Goliath’	Mixed	4.57
*Miscanthus sinensis* ‘Amur Silvergrass’	Leaf	3.83
*Miscanthus sinensis* ‘Grosse Fontaine’	Leaf	11.18
*Miscanthus sinensis* ‘Undine’	Leaf	10.27
*Miscanthus sinensis* ‘Zebrinus’	Leaf	3.94

*Abbreviations*: *RO* Root, *RZ* Spring Rhizome, *RB* Rhizome Bud, *ES* Emerging Shoot, *VA* Vegetative Shoot Apex, *SA* Sub-Apex Shoot, *ST* Stem, *PA* Pre-Flowering Apex, *II* Immature Inflorescence, *MI* Mature Inflorescence, *ML* Mature Leaf, *FR* Fall Rhizome, Mixed (RNA made after pooling RO, RZ, RB, ES, VA, SA, PA, II, MI and ML tissues);

^1^denotes 36 bp paired-end reads.

^2^denotes 76 bp paired-end reads.

^3^denotes 100 bp paired-end reads.

### Tissue specific expression profile of the *Miscanthus × giganteus* transcriptome using the *Sorghum* genome as a reference

The *M.* × *giganteus* tissues were sequenced in two separate Illumina short-read sequencing runs, both to assemble the *Miscanthus* transcriptome (Table 1, Figure 1A) and to identify genes preferentially expressed in a single *M. × giganteus* tissue-type. Approximately ten million reads were obtained for each tissue. Although *Miscanthus* does not currently have a completed genome the high nucleotide identity of *Miscanthus* to *Sorghum*[4] suggests that the *Sorghum* genome can be used as a suitable reference for profiling tissue specific transcript expression in *Miscanthus*.

**Figure 2:**
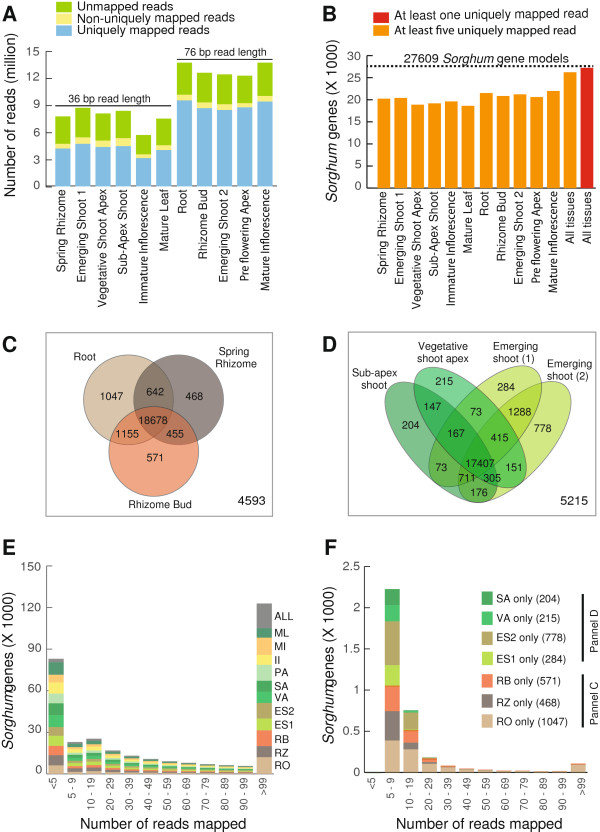
**Reads from each** ***Miscanthus*** **tissue mapped to** ***Sorghum bicolor.*** Panel **A** displays read count matching to *S. bicolor* gene models for each sequenced *M. × giganteus* tissue uniquely, non-uniquely (i.e., between two and five matches), or not at all; approximately 53% to 71% of the *M. × giganteus* reads mapped uniquely to the *Sorghum* transcripts. Panel **B** shows the number of *Sorghum* gene models represented by a minimum of five *M. × giganteus* reads for each sequenced *M. × giganteus* tissue. Panels **C** and **D** show similarities and differences in the profiles of *Sorghum* gene models represented with a minimum of five reads for select *M. × giganteus* tissues. Panel **E** shows a histogram of the total number of reads mapped per *Sorghum* gene model for each *M. × giganteus* library. Panel **F** shows the distribution of the number of reads mapped per *Sorghum* gene model in the unique categories of the Venn diagrams in panels **C** and **D**.

Reads were filtered for quality prior to their alignment to the *Sorghum bicolor* genome. Not surprisingly, more sequences were filtered from the 36 bp (base-pairs) compared to 76 bp reads. Sixty-three percent of the adapter-trimmed and quality-filtered *M.* × *giganteus* reads mapped uniquely to the *Sorghum* genome with a minimum of five *M.* × *giganteus* reads matching 26,230 of the 27,609 predicted gene models in *Sorghum* (Figure 2B). The transcript profile of each tissue typically detected about 20,000 *Sorghum* genes, ranging from 18,623 in Mature Leaf to 21,987 in Mature Inflorescence.

When expression profiles for each library are subjected to hierarchical clustering, the libraries tend to group primarily by organ type (Figure 1B). However, because some libraries were sequenced at different read lengths (36 versus 76-bp, Table 1), relative mapping efficiencies to the *Sorghum* reference could contribute to apparent relationships among libraries. We assessed this directly by three analyses. First, Figure 2A shows that libraries sequenced to 36-bp produced approximately half the proportion of reads mapping to the *Sorghum* reference compared to the 76-bp libraries. Second, when the number of *Sorghum* gene models with a minimum of five matching reads are compared among libraries from similar tissues, a substantial number of gene models appear to be uniquely represented in only one library (Figure 2C and D). This observation is particularly noteworthy for the comparison of Emerging Shoot (1, 36-bp reads) and Emerging Shoot (2, 76-bp reads), where the same RNA sample was used to independently construct two libraries. Although many *Sorghum* gene models were sampled at read depths greater than 10, a substantial number show lesser depth (Figure 2E). It is predominately these low-coverage gene models that account for the apparent differences among closely related (e.g. Vegetative Shoot Apex and Sub-Apex Shoot) or identical (e.g. Emerging shoots) RNA samples (Figure 2F).

While most transcripts are ubiquitously expressed in all tissues, transcripts that are differentially expressed yet abundant in at least one tissue are interesting as markers for developmental programs or tissue-specific biology. The Rank Products (RP) method [17, 18] is a useful non-parametric test to evaluate the significance of differential expression by a series of fold change comparisons. Rankings arise from consistencies in fold change differences between samples; as such, a series of pairwise comparisons for each individual tissue against the rest of the sequenced tissues in our study identifies high-ranking transcripts that are preferentially expressed in a tissue compared to the rest. The RP method has been used recently to help develop expression profiles for plants such as soybean [19], aspen trees [20], and the study of hormonal responses in *Arabidopsis*[21]. We employed RP to identify genes preferentially expressed in one particular tissue compared to the other sampled tissues, i.e. the “rest of the plant” (Additional file 1).

The highly ranked genes from this analysis included many whose expression is known to be associated with biological processes that occur primarily in one of the sampled tissues. Examples include photosynthetic genes like phosphoenolpyruvate carboxylase (PEPC) and pyruvate orthophosphate dikinase (PPDK) in Mature Leaf, genes involved in floral organ development like *APETALA3* and *PISTILLATA* in the Inflorescence samples, and regulators of flowering like *APETALA1* in the Pre-Flowering Apex [22–27] (Additional file 1). Overall, we believe that we have generated a good repertoire of gene expression in *Miscanthus* for a number of stages and tissues. The primary appeal of this information is its potential use in the future investigation of the *Miscanthus* genus’ unique traits and characteristics. The high rankings of genes known to be highly expressed in certain tissue types in other plant species strengthens confidence in our approach to identify genes preferentially expressed in lesser-studied organs such as the subterranean rhizome; thus, we choose to focus our validation experiments on genes preferentially expressed in the Spring Rhizome and associated organs (Rhizome Buds, Emerging Shoot and Root).

**Figure 3:**
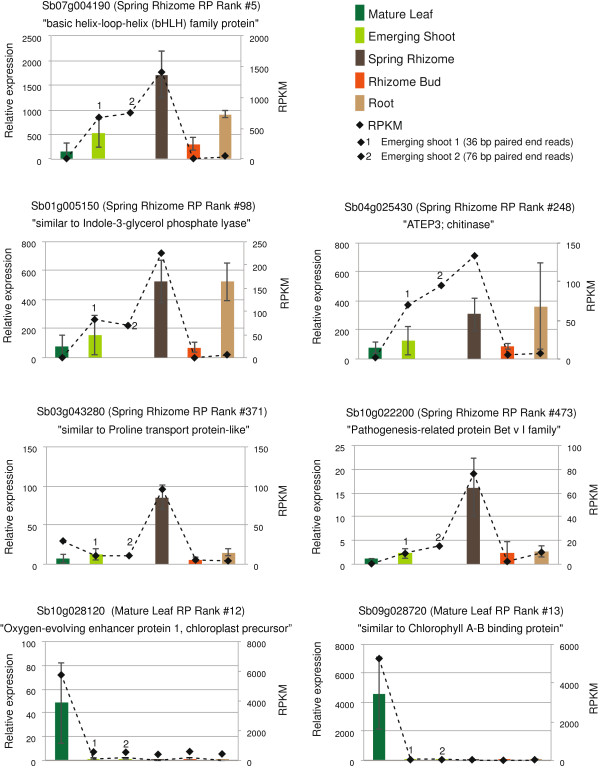
**Verification of differentially expressed genes.** Comparison of RPKM data and RT-qPCR results for five separate *M. × giganteus* tissue types. RPKM values are shown as dashed lines with values on the right y-axis. Relative expression via RT-qPCR is shown as bars with values on the left y-axis.

Five genes that showed preferential expression in the Spring Rhizome, as determined by the Rank Product analysis, were considered for verification in RT-qPCR assays. To ensure that we had independent biological replication of the samples used for RNA-Seq, new samples were collected in triplicate in Spring 2011. RT-qPCR was conducted on five tissue types from this sampling (Mature Leaf, Emerging Shoot, Rhizome, Rhizome Bud, and Root, Figure 3). These five tissues were selected based on a combination of their availability at the time of sampling in early spring, their correspondence to the tissues originally profiled via RNA-Seq, and the potentially wide range of transcript expression based upon their physiological differences from one another.

As no housekeeping genes have been tested or verified for use in *M. × giganteus,* five potential control candidates were deduced from the Rank Product data. These potential control candidates contained *Sorghum* gene models with near-equal RPKM (Reads per Kilo-base per Million) values in each of the five tested tissues used in this verification. From these five candidates, the two best-performing gene models (in terms of amplification efficiency via RT-qPCR and closest-to-equivalent expression) were chosen as control genes for this study.

The RT-qPCR results correlated well with the expression patterns estimated by the RNA-Seq analysis (Figure 3), confirming that the expression variation observed from RNA-Seq provides a good representation of changes in transcript profiles among samples. Occasionally, gene expression for the root tissue appeared higher in the RT-qPCR. We attribute this discrepancy to the differences in the growth conditions for the root tissues sampled for RNA-Seq and RT-qPCR. The RNA-Seq library was prepared from roots of greenhouse plants grown in Turface, whereas the RT-qPCR analysis was performed with root tissue harvested from the same long-standing *M. × giganteus* field plot from which the majority of other tissue samples were obtained. In addition to the aforementioned tests, two additional leaf specific genes were assayed and both methods showed consistent results (Figure 3).

### Seasonal transcription responses in *Miscanthus × giganteus* rhizomes

We noticed that a number of the genes identified by the Rank Product analysis as preferentially expressed in Spring Rhizomes were annotated with functions associated with the biosynthesis or signaling of plant hormones. Such pathways might be expected to be highly active in rejuvenating rhizomes. To assess this hypothesis more directly, we obtained biological replicate samples from rhizomes harvested in both Spring (May 5) and Fall (October 29) during the 2012 growing season and used RNA-Seq for transcript profiling (Additional file 2). A Gene Ontology analysis of these samples shows an enrichment in Spring Rhizomes of transcripts associated with cell wall biogenesis, root development, and both the biogenesis and signaling of jasmonic acid (Additional file 3). These findings confirm observations from the initial Rank Product analysis. In contrast, rhizomes collected in late fall show an enrichment of transcripts associated with seed maturation and dormancy. Overall, the upregulation of hormonal signaling in the spring and dormancy in the fall is consistent with seasonal changes in the physiological functions of rhizomes.

Quantitative trait analysis in a *Sorghum bicolor* by *Sorghum propinqum* population identified a 15 MB interval on Sorghum chromosome 1 associated with rhizomatousness and cold tolerance [28]. It is interesting to note that many genes in this interval are highly expressed in the *M. × giganteus* Rhizome and also are differentially expressed between Spring and Fall Rhizomes. Noteworthy among these genes are three predicted ZIM domain proteins (Sb01g033020, Sb01g045190, Sb01g045180) with homology to Arabidopsis JAZ/TIFY transcription factors associated with jasmonic acid biosynthesis and signaling (Additional file 2). Conversely the *M. × giganteus* homolog of Sb01g038670 is highly expressed in Fall Rhizomes (Additional file 2). Sb01g038670 encodes a putative small hydrophobic membrane protein that belongs to a low temperature and salt responsive protein family and shows similarity to Arabidopsis RCI2s and Maize PMP3s [29–33].

### De-novo assembly of the short read data

**Figure 4:**
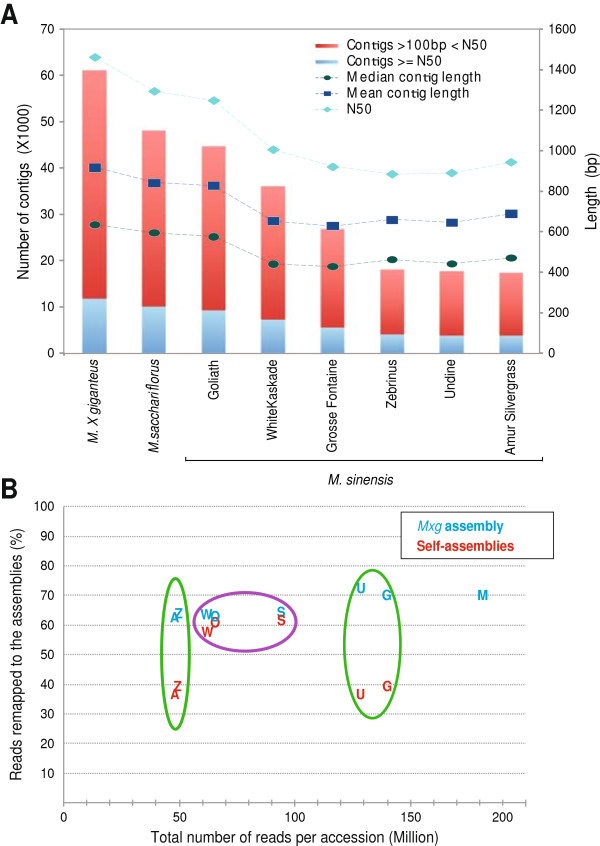
**Basic assembly statistics for the transcriptomes from eight** ***Miscanthus*** **accessions.** Panel **A** compares assembly statistics for each accession. Height of bars indicates number of contigs (left Y-axis) and lines represent length of contigs (right Y-axis). Panel **B** shows the number of reads from each accession (indicated by letters) which mapped back to either the assembly produced from that specific accession (red letters) or to the more complete assembly derived from all sequenced *M. × giganteus* libraries (blue letters). The assemblies from individual accessions where a mixture of tissues were combined into one RNA-Seq library are contained within the purple circle, whereas those assemblies derived from only leaf tissue are contained within the green circles. Mapped reads from each accessions are denoted as follows: “Z” *M. sinensis* ‘Zebrinus’, “A” *M. sinensis* ‘Amur Silvergrass,’ “W” *M. sinensis* ‘White Kaskade,’ “O” *M. sinensis* ‘Goliath,’ “S” *M. sacchariflorus* ‘Golf Course,’ “U” *M. sinensis* ‘Undine,’ “G” *M. sinensis* ‘Grosse Fontaine,’ and “M” is *M. × giganteus*.

Since a reference genome for *Miscanthus* does not exist, the sequenced short reads were assembled *de novo* using a combination of the ABySS [34] and Phrap assemblers (version 1.080721, http://www.phrap.org). Here we use “transcriptome” to refer to a collection of highly expressed genes that are deeply sampled at ample coverage for producing robust contigs (contiguous sequences) as well as low abundance genes where sequence depth and coverage limits assembly. A key parameter in assembly of short reads is the k-mer word size, which represents the minimal exact match that is needed to combine two reads into the same contig. Since low abundance genes typically assemble better with a smaller k-mer size, and highly expressed genes assemble better at larger k-mers [35], we ran ABySS multiple times using k-mer lengths between 25 and 50 bases. Following this, Phrap was used to merge the ABySS assemblies. The final *M. × giganteus* assembly contained 50,682 contigs longer than 200 bp and a contig N_50_ length of 1,459 bp (Figure 4A, ftp://ftp.jgi-psf.org/pub/JGI_data/Miscanthus/transcriptome/).

The *M. × giganteus* genotype was formed via hybridization of *M. sinensis* with *M. sacchariflorus*. Thus, we expect that the detailed assembly produced for *M. × giganteus* should also be broadly useful for investigating expression variation in other *Miscanthus* accessions. We evaluated this in two ways. First we generated libraries from a single tissue (expanding leaves containing both mature and immature portions) for four *M. sinensis* accessions and then mapped the reads to either an assembly produced only from that accession or to the *M. × giganteus* assembly. Leaf samples clearly have a reduced representation of the full transcriptome of *M. × giganteus*, as evidenced by the fewer number of contigs produced and their shorter N_50_ (Figure 4A). This is not unexpected, as most leaf tissue reads likely come from a small number of very highly expressed genes; as a result, less abundant transcripts will be more poorly represented. Importantly, when leaf-only libraries are mapped to *M. × giganteus*, the proportion of mapped reads rises to the level observed for *M. × giganteus* onto itself (Figure 4B), suggesting that nearly all reads in the leaf libraries are in fact represented within the *M. × giganteus* assembly. We reasoned there might be two approaches to improve accession-specific assemblies, greater read depth of the same tissue, or the inclusion of more tissues. Figure 4B shows that more than doubling the read depth of the leaf libraries had no impact on the proportion of mapped reads (those within the green circles); however, even a single library containing a mixture of tissues (those within purple circle) sequenced at moderate depth yields accession-specific assemblies comparable to *M. × giganteus*. Having established that moderate depth sequencing of mixed tissues offers the best assembly, we generated such a library from *M. sacchariflorus* accession ‘Golf Course’ and confirmed that the *M. × giganteus* assembly is of sufficient quality to obtain high proportions of read-mapping for both *M. sacchariflorus* and *M. sinensis* accessions.

**Figure 5:**
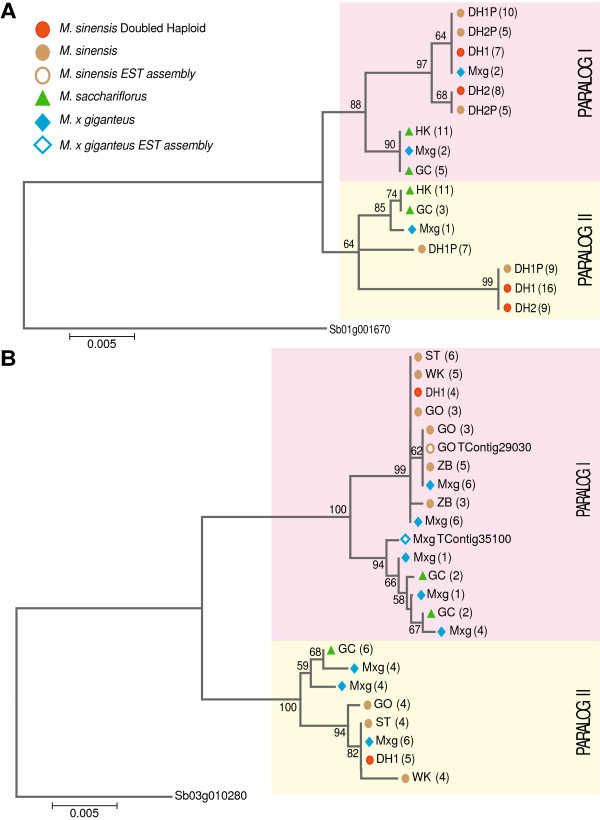
**Evolutionary relationships among** ***Miscanthus*** **gene fragments.** Maximum likelihood trees were generated for two genes; significant branches are denoted by their bootstrap value. The trees are drawn to scale, with branch lengths measured by the number of substitutions per site. Panel **A** displays a tree drawn from the alignment of a 691 bp genomic sequence homologous to Sb01g001670, which is a single copy gene annotated as a putative membrane component member of the ER protein translocation complex. Panel **B** displays a tree drawn from the alignment of a 1,097 bp exonic segment of Sb03g010280, similar to Cycling DOF Factor 1 (CDF1). The *Miscanthus* EST contigs (M × g TContig35100 and GO TContig29030) are also included in the tree. Abbreviations for accession names: *M. sinensis* ‘IGR-2011-001’ (DH1), *M. sinensis* ‘IGR-2011-002’ (DH2), *M. sinensis* ‘IGR-2011-003’ (DH1P), *M. sinensis* ‘IGR-2011-004’ (DH2P), *M. sacchariflorus* ‘Hercules’ (HK), *M. sacchariflorus* ‘Golf Course’ (GC), *M. sinensis* ‘Goliath’ (GO), *M. sinensis* ‘Silbertum’ (ST), *M. sinensis* ‘White Kaskade’ (WK), and *Miscanthus* × *giganteus* (M × g).

To verify the transcript assemblies, we selected eleven genes represented in multiple *Miscanthus* EST assemblies and amplified the genomic segments from two *M. sinensis* doubled haploid lines, DH1 (IGR-2011-001) and DH2 (IGR-2011-002), as well as their parents DH1P (IGR-2011-003) and DH2P (IGR-2011-004) [10, 36]. All eleven genomic fragments amplified successfully, demonstrating the usefulness of the assemblies. PCR fragments were then cloned and multiple clones were sequenced for each of the eleven genes using Sanger sequencing technology. An alignment of the Sanger sequences to the EST contigs confirmed that the sequence identity in the coding region was too high to consistently distinguish between the two homeologous copies solely using short reads. Therefore, it appears that the assembly reported here is often a consensus of the two paralogous gene copies. Two of these genes, Sb01g001670 and the putative flowering time regulator Sb03g010280 (Cycling DOF Factor 1), were sequenced from different *Miscanthus* accessions, including DH1 and *M. × giganteus* (Figure 5). The sequences obtained not only show clear separation of the two paralogs, but also clearly distinguish the *M. sinensis* and *M. sacchariflorus* variants within each paralogous branch (Figure 5). As expected, *M. × giganteus* carries both *M. sinensis* and *M. sacchariflorus* variants for each paralog. Furthermore, allelic variation appears evident for paralog I of Sb01g001670 within *M. sinensis* based on clear separation of two sequences derived from the likely heterozygous DH2P parent, of which only one sequence was recovered from its homozygous descendant DH2. DH1P is apparently fixed for one of these alleles.

A practical challenge of having many closely related para-alleles in *Miscanthus* spp*.* is the propensity with which chimeric products can be generated during PCR amplification due to the aberrant pairing of incompletely amplified fragments from the para-alleles during successive PCR cycles (Additional file 4). Whereas such PCR chimeras are easy to identify with Sanger sequencing of multiple clones from PCR amplicons, less rigorous methods of genotyping polyploids based on sizing of PCR-amplified fragments (e.g., SSRs) are likely to have a high error rate due to the incidence of such artifacts.

### Annotation of the *Miscanthus* assemblies

**Figure 6:**
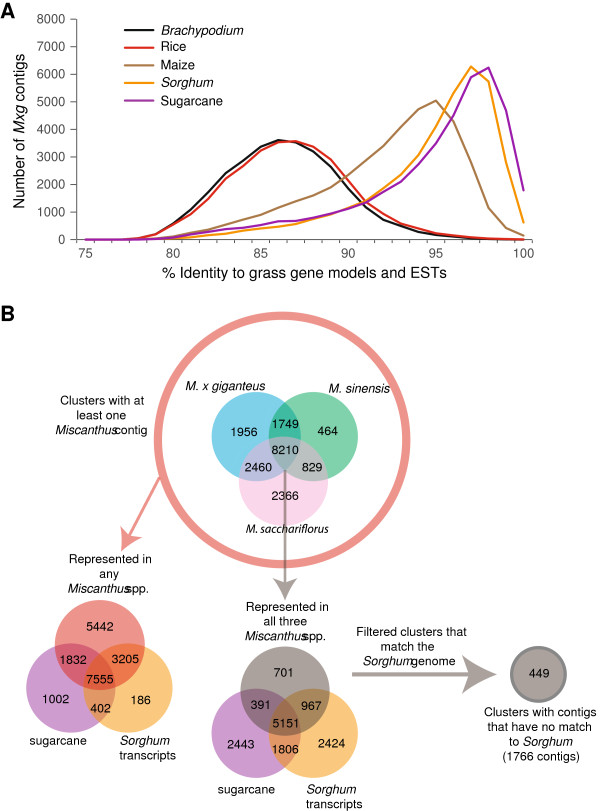
**Comparison of the assembled** ***Miscanthus*** **transcripts to gene models and ESTs of other grasses.** In Panel **A**, *Miscanthus* × *giganteus* EST contigs were compared to Sugarcane transcripts (purple), and gene models of *Sorghum bicolor* (orange), *Zea mays* (brown), *Oryza sativa* (red) and *Brachypodium* (black). The graph shows the number of contigs that match each grass transcript dataset with a given percent nucleotide identity. Panel **B** represents the clustering of *Miscanthus* contigs with *Sorghum bicolor* gene models and contigs from the Sugarcane assembled EST database (SOGI). In total, 449 clusters contain at least one *Miscanthus* contig with no match in *Sorghum bicolor* or in the SOGI database at 90% identity over 90% of its length.

The similarity of *M. × giganteus* transcripts to the gene models and ESTs of closely related grass-species *Sorghum bicolor*, *Oryza sativa* (rice), *Zea mays* (maize), *Brachypodium distachyon*, and sugarcane was assessed with a nucleotide BLAST (Figure 6A). As expected from their phylogenetic relatedness, *M. × giganteus* shows the largest degree of similarity to the sugarcane ESTs and *Sorghum bicolor* gene models, with most matches sharing over 95% identity (Figure 6A). Although the fully sequenced *Sorghum* genome is the closest comprehensive reference currently available for *Miscanthus*, the genomic and/or EST information for each of these species is potentially useful for functional annotation. The *Miscanthus* EST contigs were clustered along with *Sorghum* gene models and sugarcane ESTs using single linkage clustering. In total, 19,624 clusters were obtained; of these clusters, 8,210 have a representative from all three *Miscanthus* species. A total of 701 such clusters did not cluster with *Sorghum* gene models or Sugarcane ESTs and were studied further as putative *Miscanthus*-specific gene models (Figure 6B). This could be because the corresponding Sugarcane EST or *Sorghum* gene model is simply not present in the database or because these genes have diverged enough from their *Sorghum* and Sugarcane homologs to no longer meet the clustering conditions. Of these clusters, 449 do not share significant similarity to the *Sorghum* genome and are therefore likely to be *Miscanthus*-specific or highly divergent genes. Functional annotations are lacking for these clusters, among which 234 have no significant match (expected value <0.001) to any sequence in the non-redundant GenBank database at either the amino acid or nucleotide levels. The remaining 215 clusters match a grass sequences currently annotated as “unknowns” [37].

The *Miscanthus* contigs were annotated using InterProScan version 4.8 [38, 39]. Eighty-eight percent of contigs were assigned at least one annotation (ftp://ftp.jgi-psf.org/pub/JGI_data/Miscanthus/transcriptome/). The top twenty most common Gene Ontology (GO) assignments in the three main categories (Cellular Component, Molecular Function, and Biological Process) in the assembled *Miscanthus* transcriptome are available in Additional file 5 and provide additional evidence that we have a comprehensive collection of transcripts.

Although most repeats in the genome are silenced, it is not uncommon for some repetitive elements to show expression, particularly in actively developing tissues. Of the 269,530 *Miscanthus* contigs, 1,693 were annotated by InterProScan to contain one or more elements found in retrotransposons: Integrase, RNase H, Reverse Transcriptase and the gag structural protein (ftp://ftp.jgi-psf.org/pub/JGI_data/Miscanthus/transcriptome/). Three of these contigs (GrosseFontaine_TContig13633, Mxg_TContig47918 and Undine_TContig8294) contained all four polypeptides, suggesting they could potentially represent intact functional retrotransposons. To further investigate the presence of putative repetitive elements in the assembly, we compared the assembly to the Plant Repeat Database, which provides a comprehensive well-characterized list of the most common plant repeats [40]. Less that 2% of the contigs matched the repeat database (Additional file 6A), and more than half of these contigs were residual ribosomal RNA, likely due to incomplete removal of non-poly-adenylated RNAs during the library preparation. Aside from ribosomal RNAs, the most common matches were typically unclassified retrotransposons, transposons, and MITES of the Tourist type (Additional file 6B).

## Conclusions

The grasses of the *Andropogoneae* tribe—maize, *Sorghum*, sugarcane, and *Miscanthus*—are among the world’s most economically important crops. An abundance of genomic resources exist for the two annual crops in this group, maize and *Sorghum*. In contrast, the perennials sugarcane and *Miscanthus* have lagged behind, in part because of the size and complexity of their genomes. The *Miscanthus* transcriptome reported in this study represents a major new genomic resource for the perennial *Andropogoneae* and will enable comparative genomic studies that advance our understanding of perenniality in grasses.

This *Miscanthus* expression study provides a first glance at the transcriptome of active subterranean tissues collected during an annual seasonal cycle. It is interesting to note that these tissues show preferred expression of genes involved in jasmonic acid signaling, indole biosynthesis, auxin responses, abscisic acid pathways, and osmo-sensing. The transcripts preferentially expressed in the tissues underground suggest that changes in plant hormone pathways are associated with nutrient remobilization and growth in spring. Jasmonate synthesis and signalling appears to be particularly active in the Spring Rhizomes. Exogenous jasmonate has been shown to induce underground tubers in rhubarb, yams and potatoes, and to promote shoot and bulb formation in garlic grown via tissue culture [41–44]. It is also interesting that three ZIM/tify domain containing proteins located in the *Sorghum* rhizomatousness interval [28] are highly expressed in Spring Rhizomes while the homolog of low temperature and salt responsive protein, RCI2 [30–33, 45], in the interval is expressed in Fall Rhizomes. ZIM domain proteins are transcription factors in the jasmonic acid signaling pathway, which usually function as transcriptional repressors [46–49]. The role of jasmonate and other plant hormones in rhizome biology and nutrient cycling in *Miscanthus* deserves further investigation. In general, while hormones appear to rage in Spring Rhizomes, genes involved in amino acid metabolism and seed maturation are high in the Fall Rhizomes (Additional files 2 and 3).

As the transcriptome assembly presented here is based solely on short-read sequencing, there are situations where the paralogous transcripts are collapsed in regions of high similarity and are represented as separate contigs in regions of greater variation. It is apparent that longer read sequencing is required to produce transcript assemblies that consistently separate alleles from paralogs. Nevertheless, the information on gene expression in *Miscanthus* reported here will be valuable in exploring *Miscanthus* biology and aid in the further sequencing and annotation of the *Miscanthus* genomes.

## Methods

### Sample collection and processing

Tissue samples used in this study were collected either from a *M. × giganteus* test plot that was established in 1980 in Urbana, Illinois at the University of Illinois Turf Farm or from individual plants grown in the Plant Science Laboratory greenhouse at the University of Illinois. Specific collection information, including sampling location, tissue type, sampling time, and application are shown in Additional file 7. Root samples used in the *M. × giganteus* sequencing project were collected from rhizomes grown in the greenhouse in calcinated clay (Turface) in order to increase the efficiency of root-tissue sampling. The samples were flash frozen in liquid nitrogen immediately following their excision. Total RNA was extracted from a pool of ten biological replicates per tissue to curb the possible bias from one sample, using an RNA extraction protocol developed for pine [50]. Following the manufacturer’s protocol, Dynabeads (Invitrogen catalog number 61005) were used to purify the mRNA [51]. The yield of the mRNA was quantified with a NanoDrop Spectrophotometer ND-1000 and the quality verified on an Agilent 2100 Bioanalyzer. To ensure the highest quality possible mRNA would be used for sequencing, only samples with a 260/280 of 2 ± 0.1 and a minimum RNA integrity number of 8 were used. The libraries were made and sequenced on an Illumina Genome Analyzer IIx by the W. M. Keck Center at the University of Illinois.

For *Miscanthus × giganteus*, RNA from the various tissues was extracted and sequenced separately, with a minimum of one lane of short read data obtained for each tissue type. All samples were sequenced on an Illumina Genome Analyzer IIx. For Rhizome, Emerging Shoot 1, Vegetative Shoot Apex, Sub-Apex Shoot, Immature Inflorescence, and Mature Leaf, 36 bp paired end reads were obtained, whereas 76 bp paired end reads were obtained for the rest of the tissues. In the case of *M. sacchariflorus* ‘Golf Course,’ *M. sinensis* ‘White Kaskade,’ and *M. sinensis* ‘Goliath,’ tissues were pooled before the RNA extraction (Table 1, Additional file 7). For the rest of the *M. sinensis* accessions, expanding leaves containing both mature and immature tissues were sampled for RNA extraction and sequencing.

### Transcriptome assembly and annotation

A total of 106 billion base pairs of sequence distributed in 767 million Illumina reads were generated (Table 1, Additional file 7, SRP023501, SRP023470, SRP017791). *De novo* assemblies of the raw reads were performed separately for each accession using ABySS [34] and Phrap version 1.080721 (Phil Green, http://www.phrap.org/) as previously described in Swaminathan, 2012 [10]. Each contig was translated in all six open reading frames (ORFs) and re-oriented based on homology to a *Sorghum* gene model using BLAST, with a minimum e-value of 1E-10. If the contig showed no homology to *Sorghum*, the contig was reoriented based on the longest ORF. A FASTA file of the reoriented assembly is provided. The contigs were annotated using InterProScan version 4.8 [38, 39] Both the assembly and annotation files are available for download from ftp://ftp.jgi-psf.org/pub/JGI_data/Miscanthus/transcriptome/. The number of putative expressed repeats was identified based on homology to a repeat in the Plant Repeat Databases (ftp://ftp.plantbiology.msu.edu/pub/data/TIGR_Plant_Repeats/) using blastn with an E-value cutoff of 1E-6.

### Clustering of the contigs with the *Sorghum* annotated transcriptome and sugarcane ESTs

Single linkage [52] was used to cluster *Miscanthus* sequences with *S. bicolor* gene models [53] (ftp://ftp.jgi-psf.org/pub/compgen/phytozome/v9.0/Sbicolor_v1.4/) and sugarcane gene index (http://compbio.dfci.harvard.edu/tgi/cgi-bin/tgi/gimain.pl?gudb=s_officinarum). An all by all BLAT [54] alignment was used to find contigs that were 95% identical for over 90% of the length of the smaller of the two contigs from the same species or were 90% identical for over 90% of the length between species were assigned to the same cluster. Clusters with more than 300 members were discarded, as they are more likely to be an artifact caused by repetitive or low-complexity sequences. Clusters (701) that only contained *Miscanthus* and sugarcane sequences were re-matched to the *Sorghum bicolor* genome using Blat [54]. Clusters (449) that did not align to the *Sorghum* genome at 90% identity over 90% of the length were classified as clusters with no match to *Sorghum*.

### Cloning and sequencing genic loci from *Miscanthus* spp

Eleven genes present in a single copy within *Sorghum* were matched to the *Miscanthus* transcriptome assemblies using nucleotide-nucleotide BLAST (blastn). The best match for each gene from each *Miscanthus* assembly was aligned using Sequencher [Gene Codes Corporation version 5.0.1] with a minimum identity cutoff of 90%. Splice junctions were identified by aligning the *Miscanthus* contigs to the *Sorghum* genome using BLAT [54] with minIdentity set to 98. Thirteen primer pairs were then designed using IDT’s PrimerQuest (http://www.idtdna.com/Scitools/Applications/Primerquest/), taking care to minimize SNPs and avoid splice junctions. To confirm the primers were unique, the Novoalign program (Novoalign 2.05.13 http://www.novocraft.com/main/index.php) was used to map each primer pair to the *Sorghum* genome. The primer sequences are available in Additional file 8.

Genomic amplified PCR products were cleaned using the QIAprep Spin Miniprep kit (Qiagen catalog # 27106) and transformed using the pGem T easy Vector System II kit (Promega catalog # A1380). A minimum of eight colonies was chosen per accession for each primer; plasmids were extracted using the QIAprep 96 Turbo Miniprep Kit (Qiagen catalog # 27191). Plasmids were Sanger sequenced from both ends by the Roy J. Carver Biotechnology Center at the University of Illinois. Sequences were trimmed and aligned to the contig from which their primers were designed using Sequencher. All sequences have been deposited in Genbank (Accession numbers KF299554 - KF299740). For the two genes shown in Figure 5, genetic diversity was increased by including additional *Miscanthus* species and accessions.

Sequence ends were truncated so that every sequence was the same length; where two or more sequences from the same accession shared 100% identity, they were collapsed. Contigs were then exported in FASTA format and MEGA5 (http://www.megasoftware.net/) [55] was used for the evolutionary analyses. The evolutionary history was inferred by using the Maximum Likelihood method based on the Hasegawa-Kishino-Yano model [56], with the number of bootstrap replications set to 1,000, the number of discrete gamma categories set to five, the site coverage cutoff set at 20%, and the Close-Neighbor-Interchange set as the heuristic method.

### Expression Analysis of *Miscanthus × giganteus*

Reads were adapter-trimmed and quality controlled with Perl scripts prior to import to the CLC Genomics Workbench Version 3.7. (CLC bio 2010). Low-quality bases and bad reads were discarded from input files through the use of Trim.pl (http://wiki.bioinformatics.ucdavis.edu/index.php/Trim.pl), trimming bases with quality below 10 (phred) using windowed adaptive trimming. Reads were aligned to the unmasked *Sorghum bicolor* genome, with exon subfeatures included, downloaded from phytozome (ftp://ftp.jgi-psf.org/pub/compgen/phytozome/v9.0/Sbicolor_v1.4/), using the following settings: 94.4% identity, extend annotated gene regions 300 flanking residues both upstream and downstream, and only use reads with a maximum of five hits. Exon discovery was enabled with a required relative expression level of 0.2 with a minimum of ten reads of at least 50 nucleotides in length. Unique gene map counts were exported from CLC for each tissue file.

For the *M. × giganteus* tissue preferred expression, RPKM values were calculated based on these unique counts and subsequently used in a differential expression analysis performed via the non-parametric rank products (RP) methodology [17] using the Perl script provided by the authors. With the RP method, genes in each individual sample are ranked based on the gene-length normalized expression consistencies and differences observed when juxtaposed against the normalized expression of the other samplings by means of a series of pairwise comparisons. As a result, the final rankings for each sample identifies, preferentially expressed genes within a single tissue by comparing each tissue to all other tissue types with the exception of Emerging Shoot 1 and 2, which were treated as a single sample with expression values averaged between the two. Listings of RP results are provided in Additional file 1.

Three biological replicates of *M.* × *giganteus* were used for the Spring versus Fall Rhizome comparison. Reads were again mapped with CLC Genomics Workbench using identical parameters to those outlined above. In total, 23,015 out of the 27,609 *S. bicolor* gene models had at least one read that would map in a sample. Of these, 9,264 genes had twenty or more counts per million in at least 3 samples and were considered for the differential expression analysis using two Bioconductor packages: LIMMA and edgeR (Robinson, et al.). The LIMMA (Smyth, et al.) package was used with both FPKM (fragments per kilobase of transcript per million mapped reads) and VOOM (Law, et al.) normalization methods. A total of 3,381 genes were differentially expressed in all three methods under a false discovery rate of 0.05 and a fold change value of at least two (Additional file 2). A GO analysis was performed on the 9,264 genes using the Parametric Analysis of Gene Set Enrichment (PAGE) tool in agriGO [57] (Additional files 2 and 3).

### RT-qPCR on genes preferentially expressed in the rhizome

Total RNA was extracted from newly collected tissue-stock of *M. × giganteus* Emerging Shoot, Mature Leaf, Rhizome Bud, Root, and Spring Rhizome, all of which were sampled in April and May of 2011 from three dissimilar locations at the University of Illinois Turf Farm. Primers were designed for nine genes preferentially expressed in the rhizome according to the rank product analysis (Additional file 1, Additional file 8). For controls, five genes with near-equal RPKM expression values in each of the five sampled tissues were chosen. In addition, two primer sets for genes with known preferential leaf expression were added to this study (Additional file 1, Additional file 8). The primers were evaluated for amplification efficiency using the LightCycler Software package (ver. 1.5.0.39) on a Roche LightCycler 480. Five of the nine primer pairs designed to rhizome-preferred genes (Sb07g004190, Sb01g005150, Sb04g025430, Sb10g022200 and Sb03g043280), both the leaf genes (Sb09g028720 and Sb10g028120), and two of the controls genes (Sb09g019750 and Sb02g041180) had an amplification efficiency of 2 ± 0.1 and were chosen for RT-qPCR. As the other four of the nine primer pairs designed to rhizome-preferred genes did not possess adequate amplification efficiency, likely due to non-specific amplification, they could not be used effectively in RT-qPCR and were therefore discarded.

RT-qPCR was performed using four technical replicates and three biological replicates for every sampled tissue on a Roche LightCycler 480. Gene expression was determined by exporting data from the LightCycler Software package (ver. 1.5.0.39) into Microsoft Excel and performing a relative gene expression analysis using the ΔΔCt method [58].

### Data access and visualization

**Figure 7:**
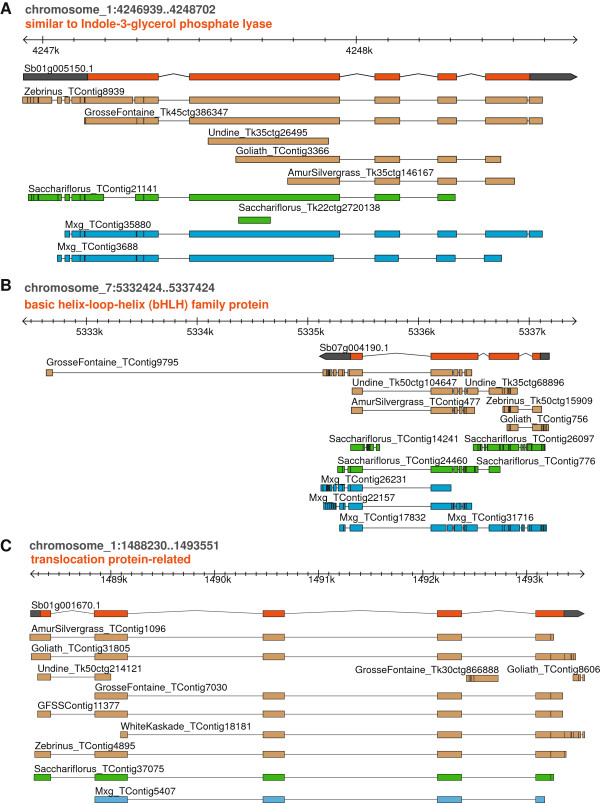
***Miscanthus*** **assemblies aligned to the** ***Sorghum*** **genome.** *Miscanthus* transcriptome assemblies aligned to the *Sorghum bicolor* genome in Phytozome. *M. sacchariflorus* contigs are shown in green, *M.* × *giganteus* contigs are in blue and *M. sinensis* contigs are brown. The *Sorghum* coding region is shown in orange and the UTRs in dark grey. The two transcripts shown in Panels **A** (homologous to Sb01g005150) and **B** (homolgous to Sb07g004190) are rhizome-preferred transcripts shown in Figure‘3. Panel **C** shows transcript homologous to Sb01g001670, which is expressed in all tissues.

The raw reads can be downloaded from NCBI’s short read archive (SRP023501, SRP023470, SRP017791). The transcriptome annotations and assemblies are available at ftp://ftp.jgi-psf.org/pub/JGI_data/Miscanthus/transcriptome/ and can be visualized at Phytozome as a track on *Sorghum* (http://www.phytozome.net/cgi-bin/gbrowse/sorghum/) (Figure 7).

## Electronic supplementary material

Additional file 1: **Tabulations of** ***Miscanthus*** **×** ***giganteus*** **reads mapped to** ***Sorghum*** **and Rank Product analysis.** (ZIP 15 MB)

Additional file 2: **Expression analysis of Spring versus Fall Rhizomes of** ***Miscanthus*** **×** ***giganteus.*** (XLSX 8 MB)

Additional file 3: **KEGG and GO term enrichment analysis of** ***Miscanthus*** **×** ***giganteus*** **Spring versus Fall Rhizomes.** (PDF 1 MB)

Additional file 4: **A chimeric sequence generated by PCR in** ***Miscanthus sinensis*** **‘IGR-2011-001’ 51 bases of the Sb01g001670 sequence showing a single chimeric clone, likely generated during the polymerase chain reaction.** Variations in the first part of the chimera match paralog II (indicated by grey arrows) while the latter part match paralog I (indicated by black arrows). The purple line shows a 103 bp region between the SNPs at positions 164 and 268, which is 100% identical in both paralogs. (PDF 2 MB)

Additional file 5: **Top 20 gene ontology terms in the 3 categories in the assembled** ***Miscanthus*** **transcripts.** (PDF 147 KB)

Additional file 6: **Characterization of** ***Miscanthus*** **contigs that match the plant repeat database.** (PDF 268 KB)

Additional file 7: **Sample collection and sequencing details.** (XLS 26 KB)

Additional file 8: **Primers used to verify gene expression and transcript assemblies.** (XLS 30 KB)
