# Supplementary material for: A detailed gene expression study of the Miscanthus genus reveals changes in the transcriptome associated with the rejuvenation of spring rhizomes
Source: BMC Genomics. 2013 Dec 9;14(1):864. doi: 10.1186/1471-2164-14-864 (PMC4046694; doi:10.1186/1471-2164-14-864)
Supplement: Supplementary file 3 — Additional file 3: KEGG and GO term enrichment analysis of Miscanthus × giganteus Spring versus Fall Rhizomes. (PDF 1 MB) [file 12864_2013_5559_MOESM3_ESM.pdf]

PLANT HORMONE SIGNAL TRANSDUCTION

Genes abundantly expressed in Spring Rhizomes compared to those in Fall, with a false discovery rate less than 0.05 and a fold change greater than 2 are colored in orange.

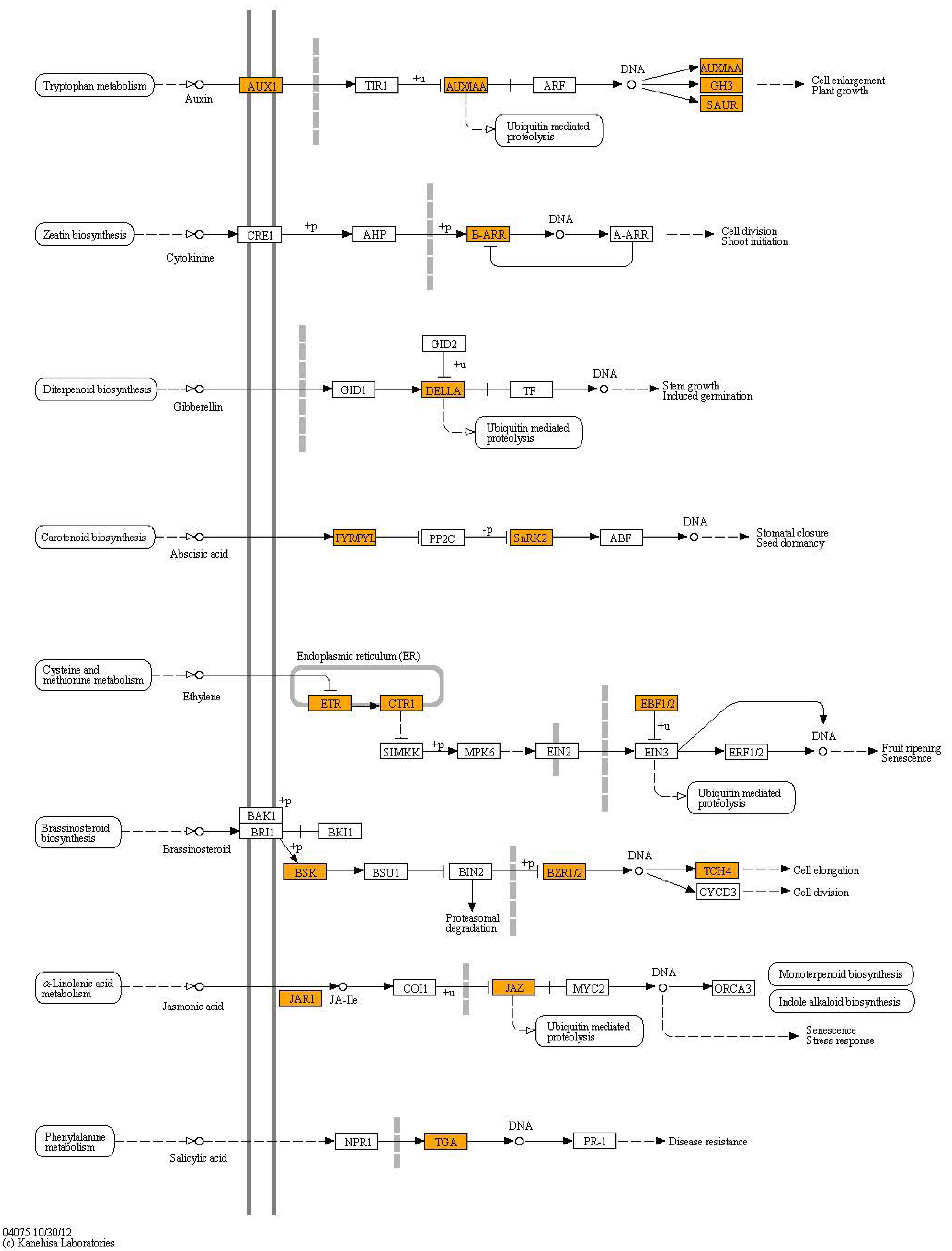

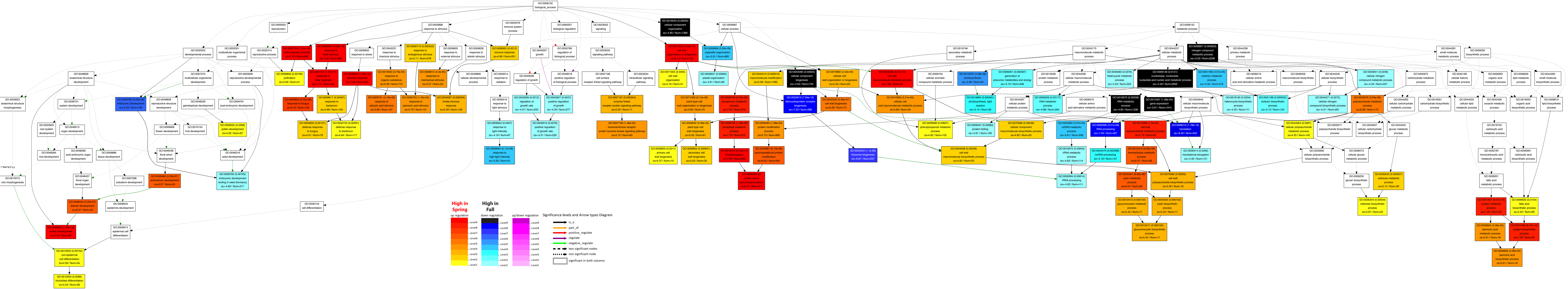
High in Spring
High in Fall
Significance levels and Arrow types Diagram
