# Supplementary figures and images for: A detailed gene expression study of the Miscanthus genus reveals changes in the transcriptome associated with the rejuvenation of spring rhizomes
Source: BMC Genomics. 2013 Dec 9;14(1):864. doi: 10.1186/1471-2164-14-864 (PMC4046694; doi:10.1186/1471-2164-14-864)

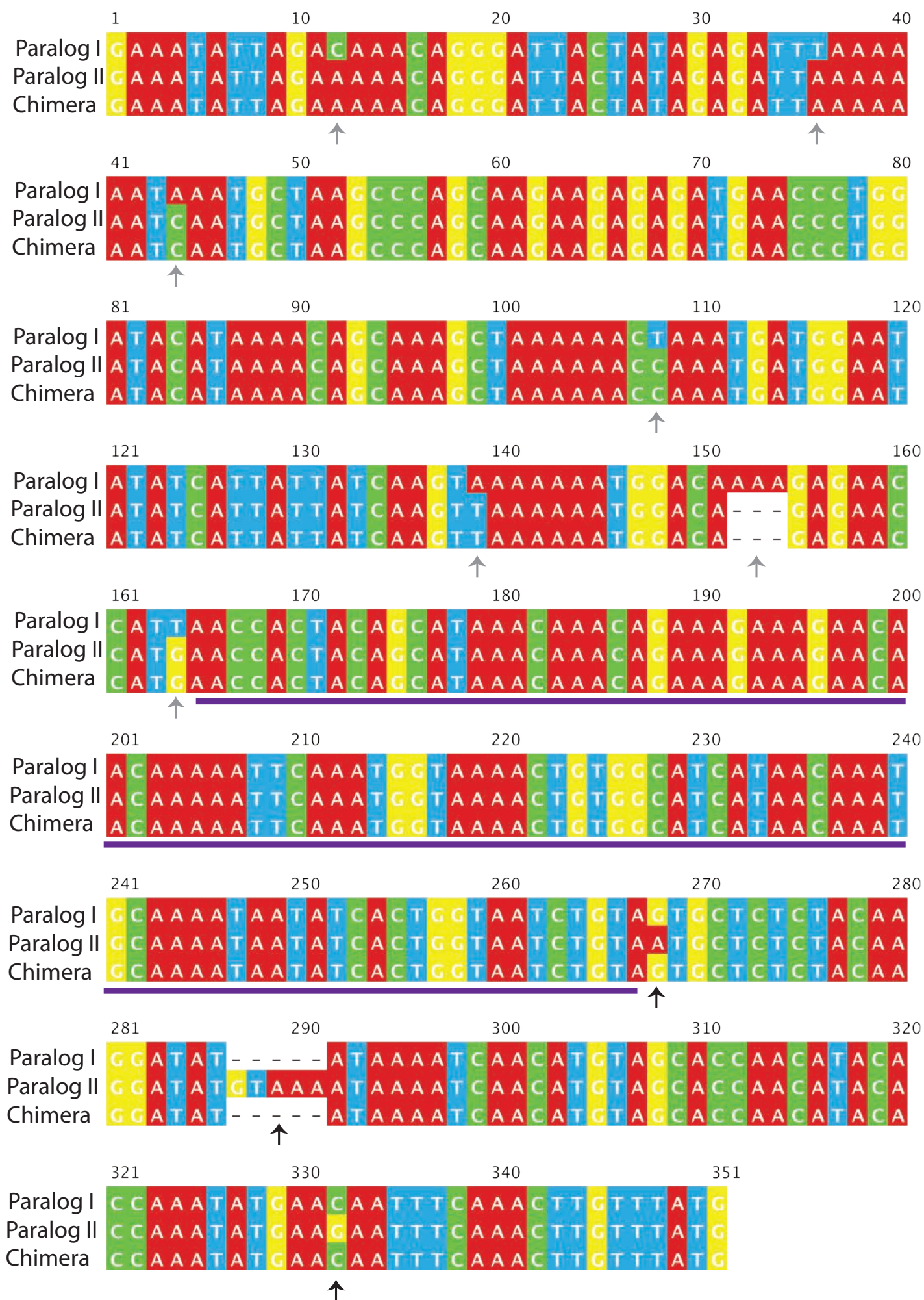

Supplement: Supplementary file 4 — Additional file 4: A chimeric sequence generated by PCR in Miscanthus sinensis ‘IGR-2011-001’ 51 bases of the Sb01g001670 sequence showing a single chimeric clone, likely generated during the polymerase chain reaction. Variations in the first part of the chimera match paralog II (indicated by grey arrows) while the latter part match paralog I (indicated by black arrows). The purple line shows a 103 bp region between the SNPs at positions 164 and 268, which is 100% identical in both paralogs. (PDF 2 MB) [file 12864_2013_5559_MOESM4_ESM.pdf]

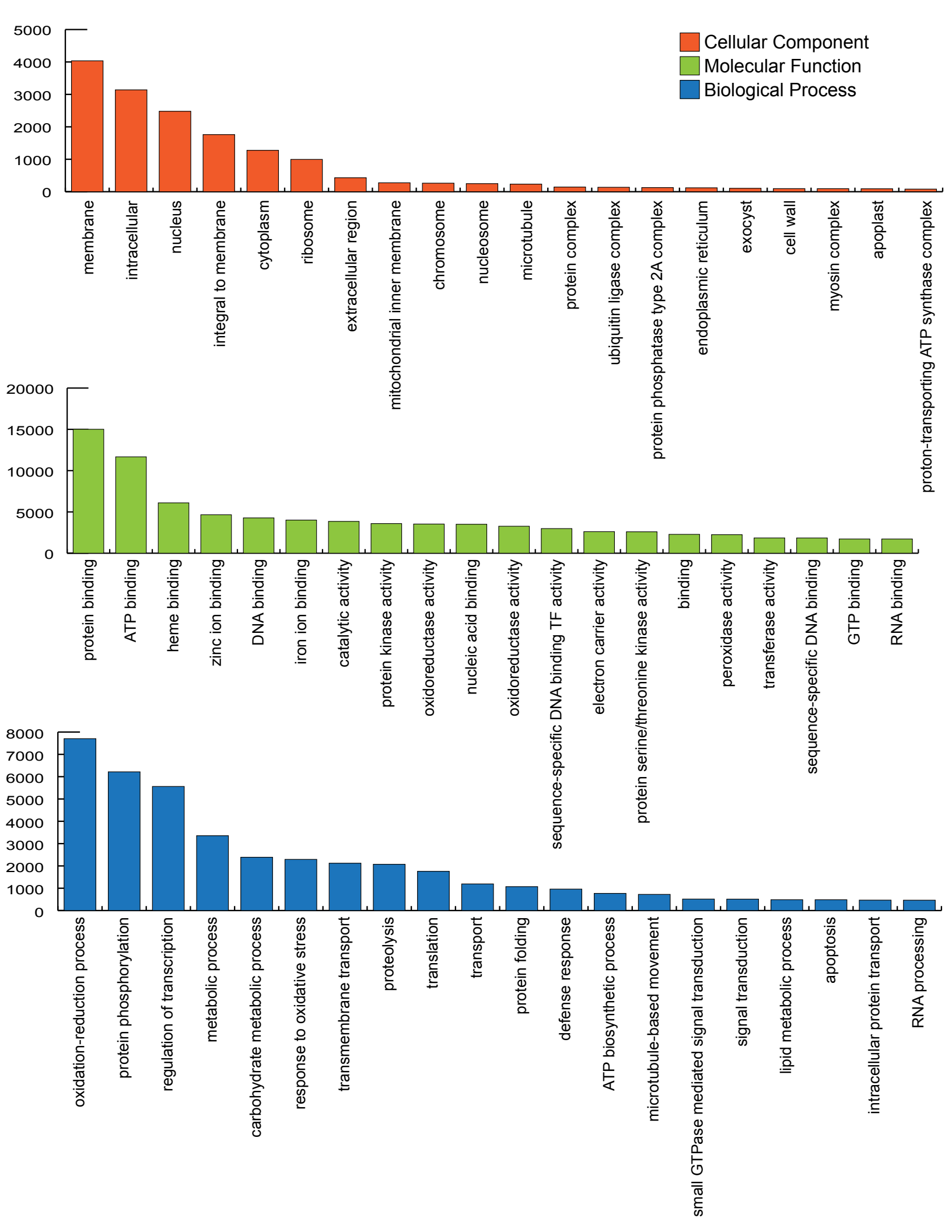

Supplement: Supplementary file 5 — Additional file 5: Top 20 gene ontology terms in the 3 categories in the assembled Miscanthus transcripts. (PDF 147 KB) [file 12864_2013_5559_MOESM5_ESM.pdf]

A

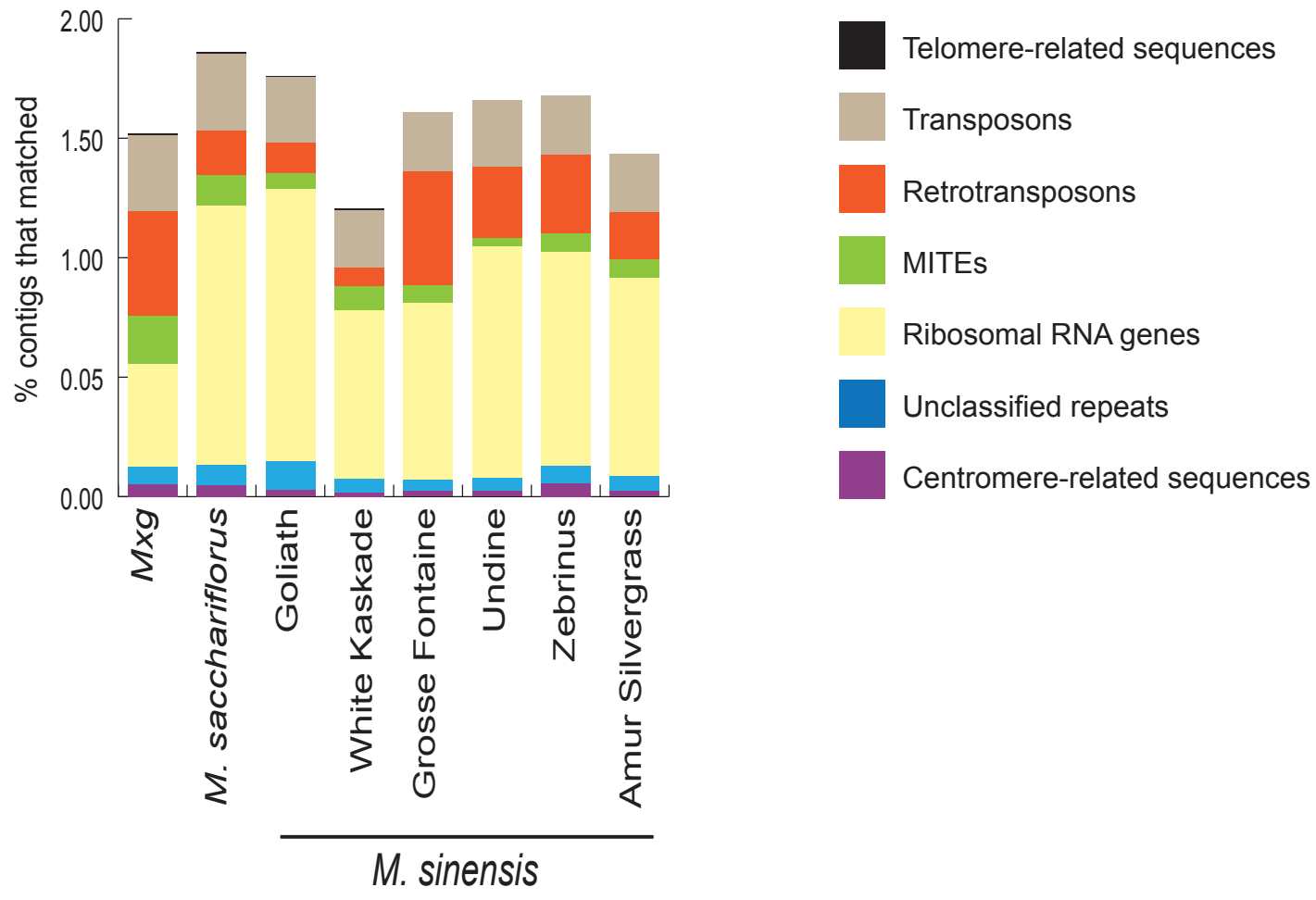

B

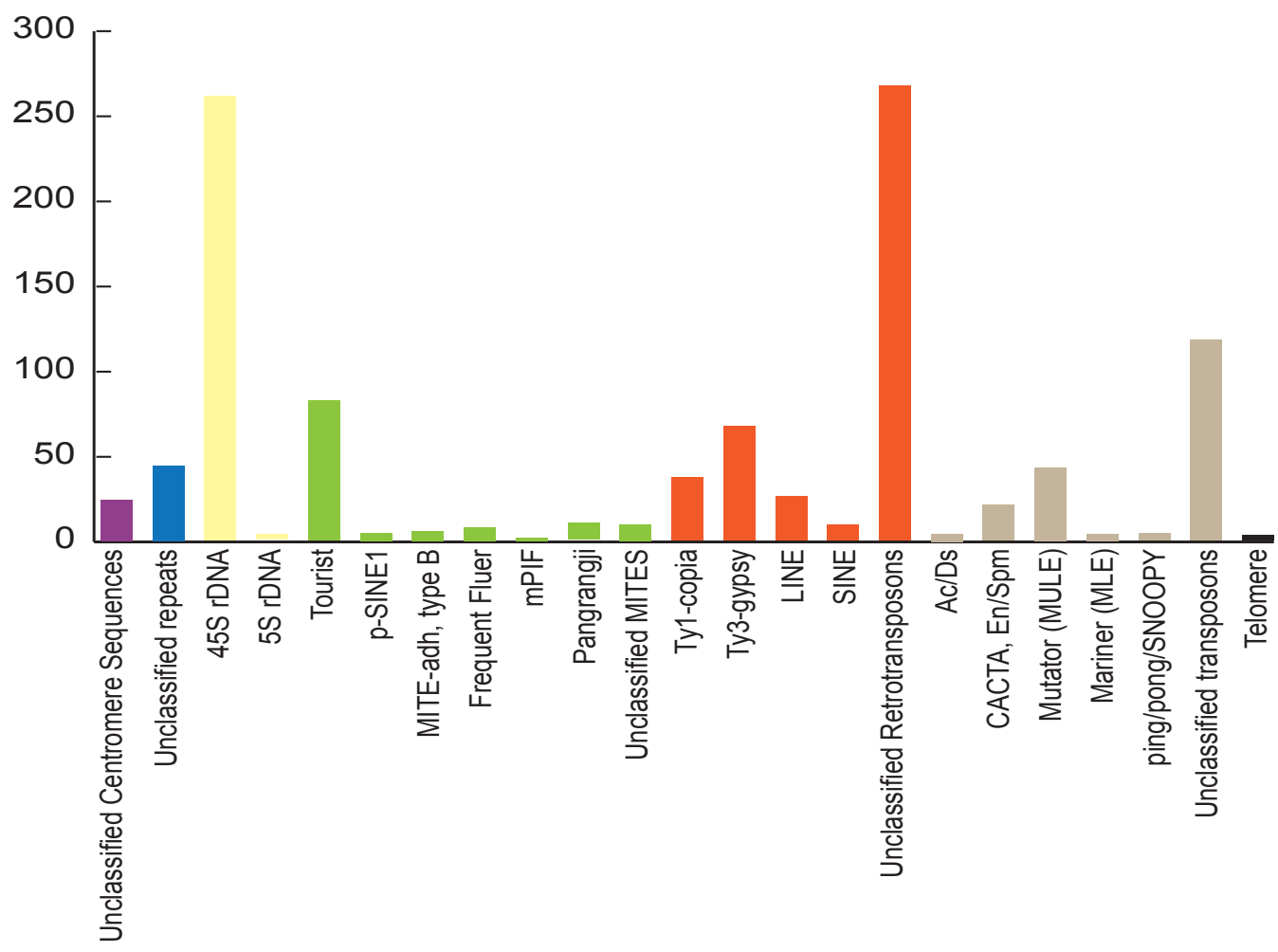

Supplement: Supplementary file 6 — Additional file 6: Characterization of Miscanthus contigs that match the plant repeat database. (PDF 268 KB) [file 12864_2013_5559_MOESM6_ESM.pdf]
